# Error mitigation in LPWAN systems: A study on the efficacy of Hamming-coded RPW

**DOI:** 10.1371/journal.pone.0304386

**Published:** 2024-06-12

**Authors:** Muhammad Moazzam Ali, Shaiful Jahari Hashim, Zaid Ahmad, Guillaume Ferre, Fakhrul Zaman Rokhani, Muhammad Akmal Chaudhary

**Affiliations:** 1 Department of Computer and Communication System Engineering, Universiti Putra, Selangor, Malaysia; 2 Laboratory for Network Modeling, Institute of Mathematical Research (INSPEM), Universiti Putra, Selangor, Malaysia; 3 Department of Electrical and Computer Engineering, COMSATS University, Islamabad, (Lahore Campus), Pakistan; 4 CNRS, Bordeaux INP, IMS, UMR 5218, Univ. Bordeaux, Talence, France; 5 Department of Electrical and Computer Engineering, Ajman University, Ajman, United Arab Emirates; Alma Mater Studiorum Universita di Bologna: Universita degli Studi di Bologna, ITALY

## Abstract

Rotating Polarization Wave (RPW) is a novel Low Power Wide Area Networks (LPWAN) technology for robust connectivity and extended coverage area as compared to other LPWAN technologies such as LoRa and Sigfox when no error detection and correction is employed. Since, IoT and Machine-to-Machine (M2M) communication demand high reliability, RPW with error correction can significantly enhance the communication reliability for critical IoT and M2M applications. Therefore, this study investigates the performance of RPW with single bit error detection and correction using Hamming codes to avoid substantial overhead. Hamming (7,4) coded RPW shows a remarkable improvement of more than 40% in error performance compared to uncoded RPW thereby making it a suitable candidate for IoT and M2M applications. Error performance of coded RPW outperforms coded Chirp Spread Spectrum (CSS) modulation used in LoRa under multipath conditions by 51%, demonstrating superior adaptability and robustness under dynamic channel conditions. These findings provide valuable insights into the ongoing developments in wireless communication systems whilst reporting Q-RPW model as a new and effective method to address the needs of developing LPWAN and IoT ecosystems.

## 1 Introduction

In recent years, there has been a growing interest in the hardware implementation of LPWAN technologies across different domains including but not limited to intelligent transportation systems, smart farming, and smart cities, etc. These technologies possess the ability to enable communication over extended distances while consuming minimal power, rendering them highly suitable for interconnecting a multitude of devices across a wide range of settings [1, 2]. The development of LPWAN technology has emerged as a feasible resolution to tackle the obstacles linked with transmitting data over long distances, minimizing power usage, and deploying IoT applications cost effectively. The utilization of LPWAN technology for Machine-to-Machine (M2M) communication facilitates monitoring of IoT devices along with their surroundings regardless of geographical location. In addition, LPWANs are considered highly suitable options for IoT applications on account for their prolonged battery longevity exceeding 10 years with extended coverage area up to a few kilometers [3, 4].

Rotating Polarization Wave (RPW) has recently emerged as a strong contender for leading LPWANs such as LoRa. The latest work by Ahmad et al. conducted a comparative link budget analysis of LoRa and RPW [5] which shows LoRa exhibits superior sensitivity and link budget compared to RPW under AWGN noise conditions, however, under fading conditions RPW surpasses LoRa in terms of bit error rate (BER) performance and range. RPW is also regarded as a superior LPWAN solution for IoT applications demanding high data rates, extended coverage, and enhanced reliability. The reliability can be improved significantly by employing error detection and correction techniques.

The studies, [6, 7] investigate the impact of Rayleigh fading on the BER of LoRa transmissions. These studies provide insights into LoRa’s performance under challenging channel conditions without exploring specific error correction or detection techniques employed within the LoRaWAN protocol itself. Instead, they focus on developing models to predict the BER under fading conditions which is crucial for understanding the inherent error resilience of LoRa communications and can inform decisions regarding the necessity for additional error correction mechanisms in specific applications. Several error correction algorithms have been utilized with LoRa depending upon the application under consideration. LoRa Forward Error Correction (FEC) coding protocol which includes fragmentation and forward error correction was suggested by Ulysse Coutaud. The LoRa communication channel is specifically engineered to provide Line of Sight (LoS) communication with a maximum range of up to 15 km. However, a significant number of parity errors may occur within this specific range. FEC algorithms utilizes Error Correction Coding (ECC) procedure to encode *M* data symbols into a codeword leading to the inclusion of N redundant symbols in the codeword. This procedure allows retrieving data-word from codeword given that the number of malformed symbols or erased data is lower which are typical characteristics of a particular error correcting code. FEC is widely used in the terrestrial and satellite communication to enhance communication reliability and data transmission capacity [8]. A thorough examination of the progress made in LoRa communication systems with a special emphasis on the incorporation of list decoding techniques to improve performance is provided in [9]. The article focused on addressing the inherent constraints of reliable communication over wide distances to acknowledge the increasing importance of LoRa technology in IoT applications. The development of LoRa technology highlighting its advantages and disadvantages is greatly discussed. The article further investigates current studies on list decoding techniques in the wider context of wireless communication networks. The performance enhancements realized through list decoding techniques were assessed using various performance metrics such as BER, Packet Error Rate (PER), and throughput under diverse channel conditions. Comparative studies demonstrated that list decoding techniques in LoRa communication exhibit higher reliability compared to traditional decoding methods. A unique error detection and adaptive error correction system built for LoRa communication, addressed the shortcomings in existing FEC approaches [10]. The proposed method significantly improves the error detection mechanism of FEC by strategically changing the original sequence using the position information of 1 bit. This enhancement is achieved through a series of stages to include a thorough examination of LoRa signal symbol defects and their patterns which involve modeling of transmitted and received signals using Fast Fourier Transform (FFT) to allow handling of mixed signals. An adaptive error correction technique to include the transmission of detection groupings for management of misregistration symbols and frequency offset data, and a confirmation procedure by probability sorting is presented. The sender transmits real data sets while, the recipient integrates findings of the enhanced error detection mechanism with an adaptive error correction system. This approach showcases improved FEC capabilities and guarantees identification and correction of every instance of an erroneous symbol. This is highly suitable for dynamic and error prone conditions found in the wireless environments. To complement recent advancements in LoRa error handling this rigorous study incorporates error detection and correction in RPW for better performance of LPWAN.

This research paper addresses the growing demand for an error detection and correction method that utilizes Rotational Polarization wave (RPW) to effectively address the complexities associated with error detection and correction in LPWAN enabled IoT devices. The contributions of this paper include ECC for reliable RPW communication, comparison between Hamming coded RP-QPSK and RP-MPSK BER under AWGN and Rayleigh fading conditions, and Hamming coded LoRa and Q-RPW BER performance. The paper comprises of Section 2 which enlists rigorous discussion on RPW signal characteristics and generation process, Section 3 details elements of RPW study with comprehensive discourse on modulation and demodulation of RPW and development of Q-RPW model and encoding and decoding of Q-RPW, Section 4 provides simulated results and discussion, and Section 5 lists the conclusion.

## 2 RPW working

The RPW is an emerging technological advancement that exhibits potential for augmenting LPWANs through its ability to facilitate more reliable M2M communication. The current literature has not thoroughly examined the concept of RPW, despite its considerable potential. This lack of research may be attributed mostly to the fact that RPW is still in its nascent phases. The researchers in [11] investigated the potential of the RPW system by developing a prototype which was subsequently subjected to a comprehensive experimental analysis. However, it is crucial to recognize that there is currently a dearth of commercially available hardware modules specifically built for RPW communication. One salient feature of RPW communication is its ability to provide deterministic communication even in situations marked by substantial disturbances. This attribute possesses notable importance inside industrial settings where transmitters and receivers are frequently immobile. In such scenarios, it has been observed that the amplitude of regularly reflected waves surpasses that of irregularly reflected waves, even in cases where there is no direct visual path connecting the transmitting and receiving entities [12]. The regular reflection of waves can be perceived as a coherent direct wave resembling the circumstances observed in classical mobile radio scenarios. The key development in the field of RPW is its adaptive modulation technique, which includes deliberately rotating the polarization of the transmitted signal. The implementation of continuous polarization rotation allows for the reception of messages at different polarization angles resulting in a received RPW signal with a 10 dB enhancement in intensity compared to a signal lacking polarization variation. The assertions pertaining to the augmentation of received signal strength and enhancement of error performance have been substantiated by field testing conducted in industrial settings, as demonstrated by K. Takei and H. Yamada [12]. The utilization of Binary Phase Shift Keying (BPSK) modulation in RPW is chosen to align with its objective of achieving resilience and maximizing the limited bandwidth resources available. The utilization of BPSK modulation has several advantages, including its simplicity in demodulation and minimal demands for channel estimate. These attributes render BPSK an appropriate modulation for real time applications. A higher order model of modulation proposed in [13] is left for further discussion in the Section 3. Because RPW is a Polarization Diversity (PD) technique, it enables development of compact and energy efficient transmitter and receiver. In addition, RPW leverages the benefits of both PD and Circular Polarized (CP) systems, hence enhancing its appeal in the rapidly developing field of IoT and M2M communication [12]. PD systems have become increasingly popular in settings with multipath propagation due to their exploitation of simple transmit and receive antenna configurations. On the other hand, findings in [14] have shown that CP systems exhibit enhanced performance in congested propagation situations compared to their linearly polarized counterparts. Challenging surroundings sometimes encompass a range of flat structures, including buildings, internal walls, and metallic creations. Such obstacles have potential to create signal reflections and multipath interference. Circular polarization offers a significant advantage in terms of its capability to restrict delay spread, and significantly reduces the negative impact of jitter and delay variation experienced in received signals [13].

**Fig 1:**
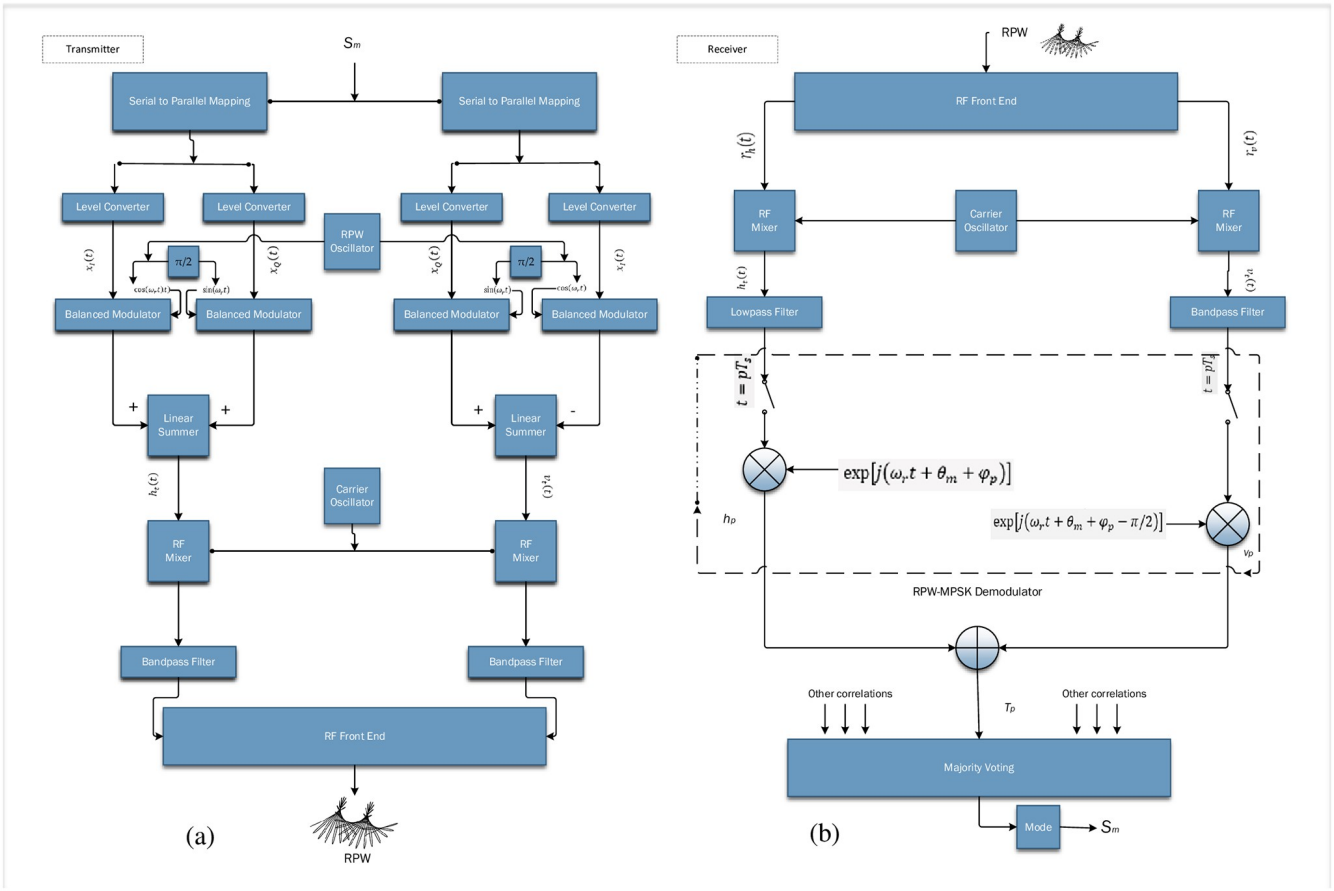
Transceiver block diagram of RP-MPSK [13].

The introduction of RPW presents an intriguing paradigm. Fundamentally, the RPW signal exhibits similar characteristics to that of a linearly polarized (LP) signal, namely at the carrier frequency *ω*_*c*_. Nevertheless, the defining characteristic of RPW is in its angular frequency, *ω*_*r*_, which operates at a much-reduced rate compared to *ω*_*c*_. The generation of this distinct signal involves the transmission of two baseband signals, each characterized by a frequency *ω*_*r*_ and a phase difference of *π*/2. This transmission occurs through a dual linear polarized antenna system operating within the carrier frequency band, *ω*_*c*_, specifically designated for Industrial, Scientific, and Medical (ISM) applications. RPW successfully combines physical characteristics of CP and PD systems, resulting in a novel solution that offers improved link dependability, especially in the context of highly reliable M2M communication networks. Fig 1 shows a representation of RPW transceiver centered at carrier frequency to provide a contrast in transmitting and receiving building blocks of the implemented RPW system. To provide clarity, let us assume that the angular velocity, denoted as *ω*_*c*_, is equal to 40*π* radians per second. Additionally, we will conservatively put the angular frequency, denoted as *ω*_*r*_, for *π*/2 radians per second. The notations for horizontal and vertical polarizations are *E*_*x*_ and *E*_*y*_, respectively. Each polarization is defined by separate amplitudes, *E*_*ox*_ and *E*_*oy*_ as given in Eq (1).

$$E\left(t\right)=E_{ox}\text{cos}\left(\omega_{r}t\right)\text{cos}{\left(\omega_{c}t+\beta_{z}\right)}_{ax}+E_{oy}\text{sin}\left(\omega_{r}t\right)\text{cos}{\left(\omega_{c}t+\beta_{z}\right)}_{ay}$$ (1)

In the current era of IoT where the interconnectedness of gadgets is fundamental to our contemporary lifestyle, the assurance of data reliability has become a crucial focal point. The network of interconnected IoT devices encompasses a wide range of disciplines, such as smart healthcare, transportation systems, industrial automation, and environmental monitoring. In various fields, the ramifications of data transmission failures can have significant impacts, encompassing decreased well-being of patients, occurrences of transportation accidents, and disruptions in manufacturing line operations. To address this difficulty, it is increasingly important to build reliable error detection and correction systems that are specifically designed for LPWAN communication. The mechanisms must include distinctive attributes of LPWAN technologies like their low power consumption and expanded range to provide optimal data reliability.

Utilization of Error Correction Codes (ECC) aims to bolster the robustness of digital systems encompassing data transfer and memory storage devices by safeguarding them against possible errors through a procedure that includes adding parity bits to the original information. This additional data helps to identify and fix any errors that may occur during storage or transmission. It should be noted that the soft errors provide a potential risk to the integrity of the data during storage or transfer procedures. These errors stem from several origins including radiation and electromagnetic interference which can lead to data corruption. Hamming codes, which are a class of error correcting codes, adds redundant bits to data to detect and correct errors during transmission in the principal form of ECC for a wide range of applications. This approach is specifically designed to add parity bits to the data to enable the identification and correction of errors. Hamming codes involves adding three extra parity bits to a set of four data bits resulting in a 7-bit code-word. The parity bits are calculated using the values of data bits and are used to detect and correct any single bit errors that may occur during storage or transmission. The use of ECC is particularly beneficial in situations that require a higher level of error tolerance such as servers, data centers, and deep space missions. Since ensuring the integrity of the data is crucial in these applications as any inaccuracies or errors can have substantial consequences, ECC offers a reliable technique for protection against soft errors to maintain accuracy and integrity of the data [15] but the type of ECC used depends on the type of application and the size of data being transmitted [4].

## 3 Study paradigm

The challenges in detecting and correcting errors reflect the innovative spirit to advance wireless communication, for better data transmission and reception in RPW supported LPWANs. In this section, the most recent modulation and demodulation techniques used in RPW together with new Q-RPW and the type of error detection and correction codes used in this study to perform error detection and correction for RPW, and development of Hamming coded software are comprehensively discussed.

### 3.1 Modulation and demodulation

The regression in error performance can be effectively mitigated by implementing a greater sampling rate at the receiver which allows for the receipt of signals across a broad spectrum of polarization angles. The increased variety of polarization angles offers a wider range of choices for selecting polarization throughout the process of detecting the message signal. The incorporation of higher order modulations involving message signal entails increased intricacy in the designs of both the transmitting and receiving systems. In comparison to PD systems, which require just one modulator, the design of a RPW transmitter is very complex which involves the utilization of two distinct and non identical Phase Shift Keying (PSK) modulators [16]. It is worth mentioning that RPW ensures signal integrity in varying propagation environments through precise control of polarization states. The previous literature on RPW has mostly focused on the investigation of RP-BPSK, RP-QPSK and RP-MPSK modulation schemes [13, 16]. RPW of a waveform is achieved by transmitting two baseband signals with a phase difference of *π*/2 through two orthogonal polarizations. This causes the polarization of the signal to rotate at a frequency of *ω*_*r*_. On the receiver end the received RPW signal is captured by a separate DP antenna and is subjected to sampling at a sampling frequency *f*_*s*_ which is an integral multiple of *f*_*r*_.

The foundational principles of classical modulation theory have been widely acknowledged for Quadrature Phase Shift Keying (QPSK) which exhibit superior performance compared to BPSK in terms of spectral efficiency. QPSK enables higher data rates while simultaneously ensuring an equivalent BER performance to that of BPSK as supported by reference [16]. The recognition of this knowledge led to the investigation of Rotating Polarization Quadrature Phase Shift Keying (RP-QPSK) a modulation technique designed to use the benefits of QPSK in the domain of RPW communication. Nevertheless, the adoption of RP-QPSK presented a substantial obstacle including both QPSK symbols and two orthogonal polarized signals with a 90° phase difference on the carriers simultaneously have proved to be a significant challenge. This limitation arose due to the inherent constraints associated with using two identical carriers for RP-BPSK. To achieve effective implementation of RP-QPSK, two QPSK transmitters were required to run in synchronization on the identical baseband frequency facilitating the production of the separate RPW signals. The exploration of the complexities involved in the advancement of RP-QPSK emerged as a crucial area of focus aiming to integrate the advantages of QPSK’s spectral efficiency with the distinguishing attributes of RPW communication. Each sample acquired through this method on the two antennas exhibits polarization at a distinct angle and serves as a duplicate of the broadcasted symbol. The desired sample is selected by choosing the signal with higher signal power between the two polarizations for each sample. Subsequently, every chosen sample is demodulated to retrieve an exact copy of the broadcasted symbol. The Eq (2) explains the horizontal and vertical polarized signals for RP-QPSK.

$$\begin{array}{c} \begin{array}{c} h_{t}\left(t\right)=x_{e}\psi_{1}\left(t\right)+x_{0}\psi_{2}\left(t\right)\\ v_{t}\left(t\right)=x_{e}\psi_{2}\left(t\right)-x_{0}\psi_{1}\left(t\right) \end{array}\}\; \end{array}$$ (2)

Where *ψ*_1_ and *ψ*_2_ are the orthogonal basis functions defined by: $\psi_{1}\left(t\right)=\sqrt{2/T_{r}\text{cos}\left(\omega_{r}t\right)}$ and $\psi_{2}\left(t\right)=\sqrt{2/T_{r}\text{sin}\left(\omega_{r}t\right)}$

It should be noted that the positions of even and odd bits have been interchanged in the signals intended for transmission via horizontal and vertical polarizations. RP-QPSK can now be represented in a more comprehensive manner in Eq (3).


$$\begin{array}{c} \begin{array}{c} h_{t}\left(t\right)=\sqrt{2E_{s}/T_{r}}\text{cos}\left(\omega_{r}t+\left(2m+1\right)\pi /4\right)\\ v_{t}\left(t\right)=\sqrt{2E_{s}/T_{r}}\text{sin}\left(\omega_{r}t+\left(2m+1\right)\pi /4\right) \end{array}\}\;m\in 0,1,2,3 \end{array}$$ (3)

Examining the performance of BER in RPW communication brings up the noteworthy observation that the use of RP-QPSK instead of RP- BPSK does not have a negative impact on BER performance. The primary factor to be considered is that achieving an improved data rate is feasible albeit with a trade-off in terms of BER and energy efficiency however, this trade-off can be overcome. To augment the data rate and energy efficiency of communication based on RPW an approach known as Rotating Polarization Multilevel Phase Shift Keying (RP-MPSK) modulation has been reported in [13] which provides higher data rates and energy efficiency. While RPW offers advantages in terms of reliable data transmission in challenging environments, its energy consumption characteristics require further investigation. The potential for lower power operation compared to existing LPWAN solutions like LoRa stems from RPW’s utilization of polarization diversity. This diversity allows the signal to potentially overcome noise and fading effects, enabling transmission at lower power levels for similar communication ranges. Additionally, research suggests that employing higher-order modulation schemes like MPSK within the RPW framework holds promise for achieving higher data rates. However, this needs to be balanced against potential increases in bit error rate. If the receiver can sample the higher-order modulated signal at a sufficiently high rate, it might be possible to transmit more data with comparable BER performance, effectively translating to lower energy consumption per transmitted bit [13]. The primary factor to be considered is that achieving an improved data rate is feasible albeit with a trade-off in terms of BER and energy efficiency however, this trade-off can be overcome. Hence, the integration of RP-MPSK modulation with enhanced receiver sampling rates presents a potential solution for establishing a reliable and effective communication system. This approach enables the utilization of greater data rates while ensuring the preservation of integrity of the transmitted information.

The generation of RPW requires a setup within the transmitter, as illustrated in Fig 1(a). The proposed configuration involves equipping the transmitter with two separate baseband phase shift keying modulators each functioning at a frequency of *ω*_*r*_ = 2*π* = *f*_*r*_ = 2*π*/*T*_*r*_. The modulators play a critical role in maintaining orthogonality between them, with one dedicated to horizontal polarization and the other to vertical polarization. The primary element of this procedure entails the concurrent entry of the data symbol *s*_*m*_ into both modulators. The process of simultaneous modulation yields two separate signals namely *h*_*t*_(*t*) and *v*_*t*_(*t*), where the subscript “t” denotes their association with the transmitted signal. The process involves the establishment of a fundamental framework for the encoding of information contained within the data symbol *s*_*m*_ onto orthogonal polarizations namely horizontal *h*_*t*_(*t*)and vertical *v*_*t*_(*t*). RPW communication employs polarized signals transmission leveraging spatial diversity from orthogonal polarizations, thus enhancing data transfer scope and communication system reliability with its utilization for forming the core of RPW’s distinctive features and capabilities [13]. The RP-MPSK transmitter is similar to the RP-QPSK transmitter however there are differences in the serial to parallel logic when using a higher level of modulation. Fig 1(b) is a block diagram illustrating the digital implementation of the RP-MPSK receiver. The received signals on the two elements of a dual polarized antenna experience significant degradation due to scattering multipath propagation. Both regular and irregular reflections are given in Eqs (4) and (5).

$$\begin{array}{c} \begin{array}{c} r_{h}\left(t\right)=h_{t}\left[t\right]\sum_{n}a_{n}\text{cos}\varphi_{n}\text{cos}\left(\omega_{c}t+\theta_{n}\right)+n_{h}\\ r_{v}\left(t\right)=v_{t}\left[t\right]\sum_{n}b_{n}\text{sin}\varphi_{n}\text{cos}\left(\omega_{c}t+\theta_{n}\right)+n_{v} \end{array}\}\; \end{array}$$ (4)

Received base-band signals are sampled at frequency $f_{s}=1/T_{s}=N_{p},f_{r}\|f_{c},N_{p}\in Z$. Each sample is then coherently demodulated using Eq (5):

$$\begin{array}{c} \begin{array}{c} h_{p}=h_{r}\left(pT_{s}\right)\text{cos}\left(\omega_{r}pT_{s}+2p\pi /N_{p}\right)\\ v_{p}=v_{r}\left(pT_{s}\right)\text{sin}\left(\omega_{r}pT_{s}+2p\pi /N_{p}\right) \end{array}\}\;p\in [0,1,2,...,N_{p}-1] \end{array}$$ (5)

In view of forgoing detailed exposition of RPW this research study employs altered RPW to describe polarization states of electromagnetic waves using quaternion mathematics involving the use of quaternion algebra to represent rotations in three-dimensional space. Quaternion symbols for dual polarized system are represented *Q*_*m*_ = *s*_*H*_ + *s*_*V*_*j*. Rotation of *Q*_*m*_ in polarization domain by an angle of *φ*_1_ can be represented as *Q*_*m*,*l*_ = *Q*_*m*_*e*^−*jφl*^. For Q-RPW transmission *Q*_*m*_ is rotated by *N*_*l*_ polarization angles to generate *Q*_*m*,*l*_ such that $Q_{m,l}=Q_{m}e^{-j2l\pi /N_{l}}l=0,1,2,\ldots ,N_{l}-1$ are obtained. From an implementation perspective this is equivalent to interpolating the transmitted symbol stream *Q*_*m*_ by factor of *N*_*l*_.

### 3.2 Encoding and decoding of Q-RPW

In the constantly evolving world of modern communication systems the integration of emerging technologies like the IoT and LPWAN with novel signal modulation techniques particularly RPW offers a promising opportunity to enhance the effectiveness and dependability of information transmission. This study undertakes a thorough investigation of the encoding and decoding mechanisms of RPW specifically within the framework of LPWAN and IoT applications. This study probes into the synergies between the distinctive qualities of RPW the energy efficient capabilities of LPWAN, and the different requirements of IoT devices with a specific emphasis on the integration of Hamming codes as a robust error correction mechanism. Additionally, for applications primarily related with single-bit error correction and where codeword size is a limiting factor, Hamming codes offer superior efficiency. They achieve this correction with less redundancy compared to Reed-Solomon (RS) codes which are designed to handle multiple errors within a codeword [4]. The robust error correction capabilities of RS codes involve complexity to offer advantages in channels with significant noise or for applications transmitting large data packets where correcting multiple errors within a codeword is crucial. While RS codes offer potent error correction, Hamming codes present a compelling alternative in specific scenarios due to their inherent advantages. The well-defined decoding structure of Hamming codes, exemplified by syndrome decoding, translates into lower implementation complexity and low memory footprint compared to RS codes that rely on algorithms like Berlekamp-Massey [17]. This is particularly beneficial in resource-constrained environments like wireless sensor networks, where low-complexity decoding is essential [18, 19]. Besides, the simpler decoding process of Hamming codes results in lower latency, a crucial factor in real-time applications.

Since society increasingly relies on networked devices for various tasks, understanding the complexities of encoding and decoding in the RPW system is of utmost importance. This understanding is crucial for enhancing communication reliability and security inside LPWAN enabled IoT ecosystems. The utilization of Hamming codes in the encoding and decoding procedures within communication systems has a significant benefit in relation to power efficiency when compared to alternative error correction codes. Hamming codes are a type of block codes that exhibit the unique capability of detecting and correcting single bit errors [20]. This property renders them highly suitable for scenarios where power conservation is of vital importance.

In the same vein, the simple structure of Hamming codes reduces hardware complexity, this potentially lowering the power consumption [21]. The strategic use of Hamming codes in low-noise environments can achieve reliable data transmission with less power than more complex methods [22]. The study in [23] positioned Hamming codes as a potential standard due to their balance between power consumption and error correction capabilities. Conclusively, the combination of simple hardware implementation, strategic application in low-noise scenarios, and potential as a low-power design baseline makes Hamming codes an asset in achieving energy efficiency. The utilization of Hamming codes in IoT devices and LPWAN environments is appealing due to their capacity to offer fault resistance without significantly impacting computational resources, which is particularly advantageous considering the inherent limitations on power consumption [24]. The efficient application of power in Hamming codes corresponds to the larger objectives of sustainability and energy efficiency in contemporary communication systems. Hamming codes play an important role in reducing power consumption by decreasing the computational cost related to error correction. This contributes to the durability of battery powered devices and facilitates the seamless integration of IoT inside LPWAN architectures [25, 26]. The objective of this study is to examine the intricacies of power efficient encoding and decoding techniques by specifically focusing on Hamming codes within the domains of LPWAN and IoT, thereby contributing to the existing research on communication technologies that prioritize energy conservation. The Hamming code is widely employed as an ECC renowned for its ability to detect and correct single bit errors. The codeword of the Hamming code with parameters (*n*, *k*) is composed of n bits divided into two parts. The k bits whose indices are not powers of two bits represent the message bits while the remaining n—k bits are referred to as parity check bits. These additional bits serve the purpose of detecting and correcting single bit errors. The Hamming (7, 4) code which is a widely used Hamming code system for a 7-bit codeword of four message bits and three parity-check bits is applied in this research study for encoding and decoding of RP-MPSK and RP-QPSK. In the following illustration, a demonstration of the encoding process of the Hamming (7, 4) code is presented wherein four message bits are transformed into a single 7-bit codeword. As an example: one block of codeword made from 100,000 symbols with parity bits is shown in Table 1, where P1, P2, and P4 are the parity bits and D3, D5, D6, and D7 are the data bits.

**Table 1:** Example of one block of codeword.

P1	P2	D3	P4	D5	D6	D7
**1**	**1**	0	**1**	0	0	1
**0**	**1**	0	**1**	0	1	0
**1**	**0**	0	**0**	0	1	1
**1**	**0**	0	**1**	1	0	0
**0**	**1**	0	**0**	1	0	1

Additionally, the code possesses the capability to rectify a single bit error within the codeword. To perform its operations, the Hamming (7, 4) code requires precomputation of two matrices: the code generator matrix, denoted as G, and the parity check matrix, denoted as H. An illustration of the two matrices adopted for this research study is presented as follows:

$$G=[\begin{array}{cccc} 1 & 1 & 0 & 1\\ 1 & 0 & 1 & 1\\ 1 & 0 & 0 & 0\\ 0 & 1 & 1 & 1\\ 0 & 1 & 0 & 0\\ 0 & 0 & 1 & 0\\ 0 & 0 & 0 & 1 \end{array}]\;H=[\begin{array}{ccccccc} 1 & 0 & 1 & 0 & 1 & 0 & 1\\ 0 & 1 & 1 & 0 & 0 & 1 & 1\\ 0 & 0 & 0 & 1 & 1 & 1 & 1 \end{array}]$$

The mathematical expression to encapsulate the encoding procedure for Hamming (7,4) codes is as follows: *CD* = mod((*data*)*G′, 2). In this expression, “data” represents the original data block that is to be communicated, and G denotes the generator matrix of the Hamming (7,4) code. Within this particular scenario, the generating matrix G plays a pivotal role in enabling the conversion of the initial 4-bit data block into a 7-bit encoded block. Matrix multiplication involves the computation of the dot product between a data block and the transpose of a generating matrix G’. The codeword (CD), which is a 7-bit vector, is designed to include strategically placed redundant parity bits to enable the identification and rectification of errors. The modulo-2 operation is thereafter employed to ensure that the encoded block remains confined within the binary field. The encoding procedure has a twofold function: (1) to improve the resilience of the transmitted data against errors, and (2) to facilitate the subsequent decoding process in detecting and correcting possible inconsistencies. The Hamming (7,4) code, characterized by its systematic architecture, serves as a notable illustration of a proficient method for error detection and correction. Consequently, it offers significant benefits in scenarios where ensuring the integrity of data is of great importance. In order to retrieve the message from Hamming (7,4) code, syndrome decoding employed which is applicable to a wide range of Hamming code parameters. This eliminates the need for code specific decoding logic by being, particularly advantageous for variable parameter or dynamic code selection. Additionally, syndrome decoding possesses the potential to detect not only single-bit errors but also double errors in some code configurations a crucial feature for noisy channels susceptible to multiple errors. The systematic mathematical structure of syndrome decoding facilitates analysis, debugging, and potential optimization compared to custom logic tailored for specific codes. However, for fixed-parameter codes like (7,4), custom logic utilizing bit wise operations can achieve superior efficiency in terms of memory and computation compared to syndrome decoding, which might involve less efficient matrix operations. Custom logic can also offer greater simplicity and ease of implementation, especially for less complex codes. Ultimately, the selection between syndrome decoding and custom logic hinges on the specific application’s requirements. If flexibility and robust error detection are paramount, syndrome decoding remains the preferred choice which is why it has been considered in this research study. Conversely, for applications with fixed codes prioritizing efficiency and simplicity, custom logic can be a more suitable option [17].

The process of decoding messages encoded with the Hamming (7,4) code involves a meticulous series of steps aimed at identifying and rectifying errors introduced during transmission. The process involves dividing the encoded message into segments consisting of 7 bits each. For every segment, a syndrome is determined by performing matrix multiplication with the predetermined parity-check matrix H. The matrix shown above has been specifically designed for the Hamming (7,4) code, with the purpose of efficiently detecting errors occurring within the block. The derived syndrome is further examined to identify the precise location of any existing errors. If an error is identified, the algorithm rectifies it through the process of toggling the bit that corresponds to the defect. Afterwards, the relevant components (except data bits) are removed from the block and added to the developing decoded message. The iterative nature of this procedure guarantees a systematic approach to address errors throughout the whole encoded message. As a result, a decoded message is obtained to accurately reflect the original data, while also minimizing the effects of potential transmission errors.

**Fig 2:**
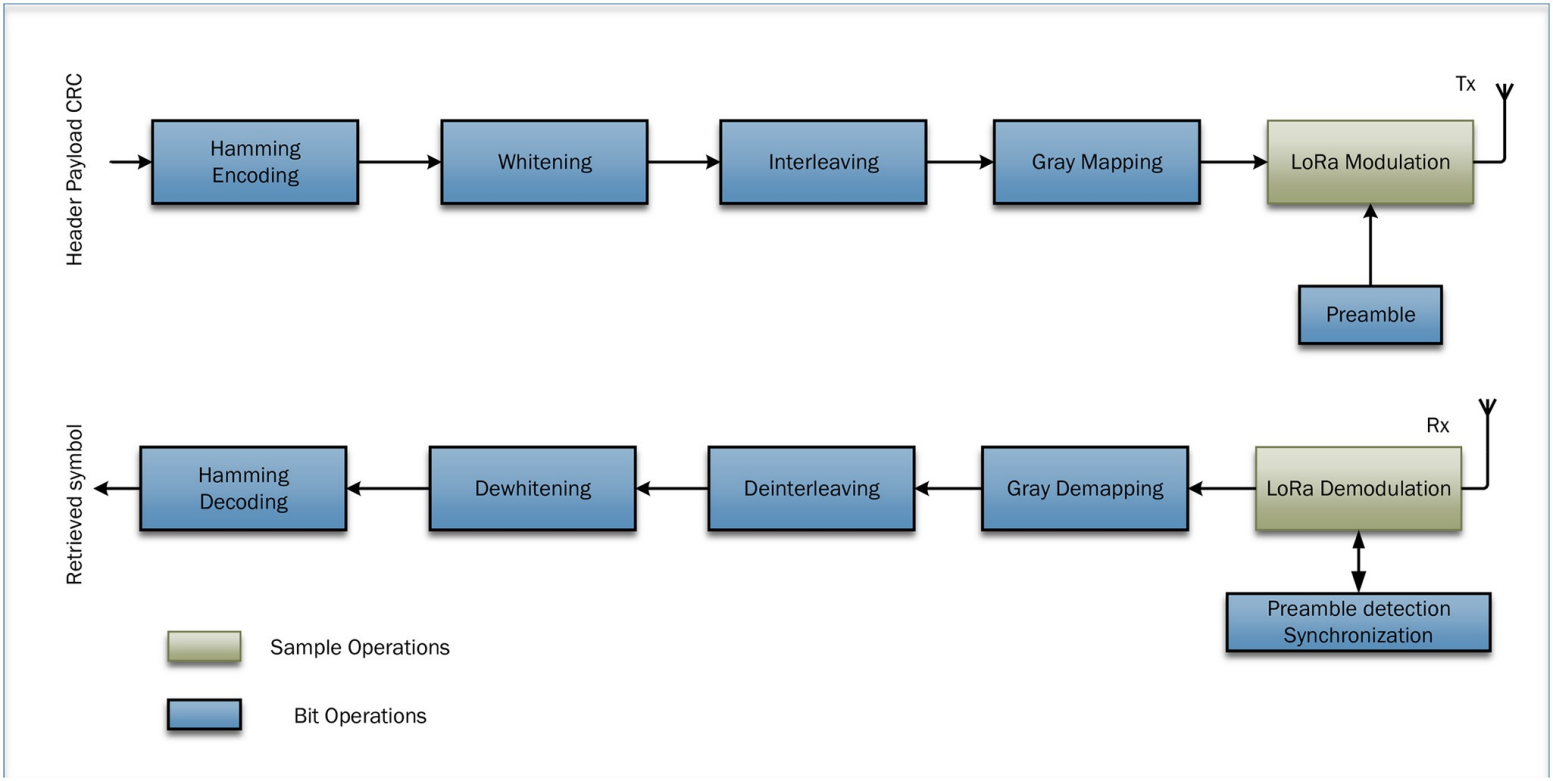
LoRa PHY based Transmitter (Tx) and Receiver (Rx) [27].

The study conducted in [27] provides a detailed investigation into the frame error rate (FER) of Hamming Coded LoRa modulation when subjected to additive white AWGN and carrier frequency offset (CFO). The study used well established approximations to determine the BER of LoRa in the presence of AWGN as shown in Fig 2.

The analysis is expanded to incorporate the effects of channel coding, interleaving, and gray mapping in the LoRa physical layer. The authors also calculated the BER of LoRa under CFO and provided a related study of the FER. To verify the precision of its conclusions, the study utilized Monte Carlo simulations and contrasted the outcomes with the derived frame error rate expressions. The authors provided a concise summary of their main contributions which involved developing simplified methods for estimating the error rate of coded LoRa signals in the presence of AWGN. The second approximation showed a high degree of agreement, with a deviation of only 0.2 dB, when compared to Monte Carlo simulations conducted at different spreading factors and FERs as low as 10^−5^. The study further emphasized the resilience of LoRa to low residual CFO values, under scoring the beneficial effects of gray mapping, interleaving, and coding. The research finishes by presenting a simplified method for estimating the LoRa FER under CFO. This estimate demonstrates a minimal divergence of 0.5 dB or less compared to the results obtained from the equivalent Monte Carlo simulation. The research offers a thorough comprehension of the encoded FER performance of Hamming Coded LoRa in difficult communication conditions providing useful knowledge for future improvements in wireless communication and IoT applications.

**Fig 3:**
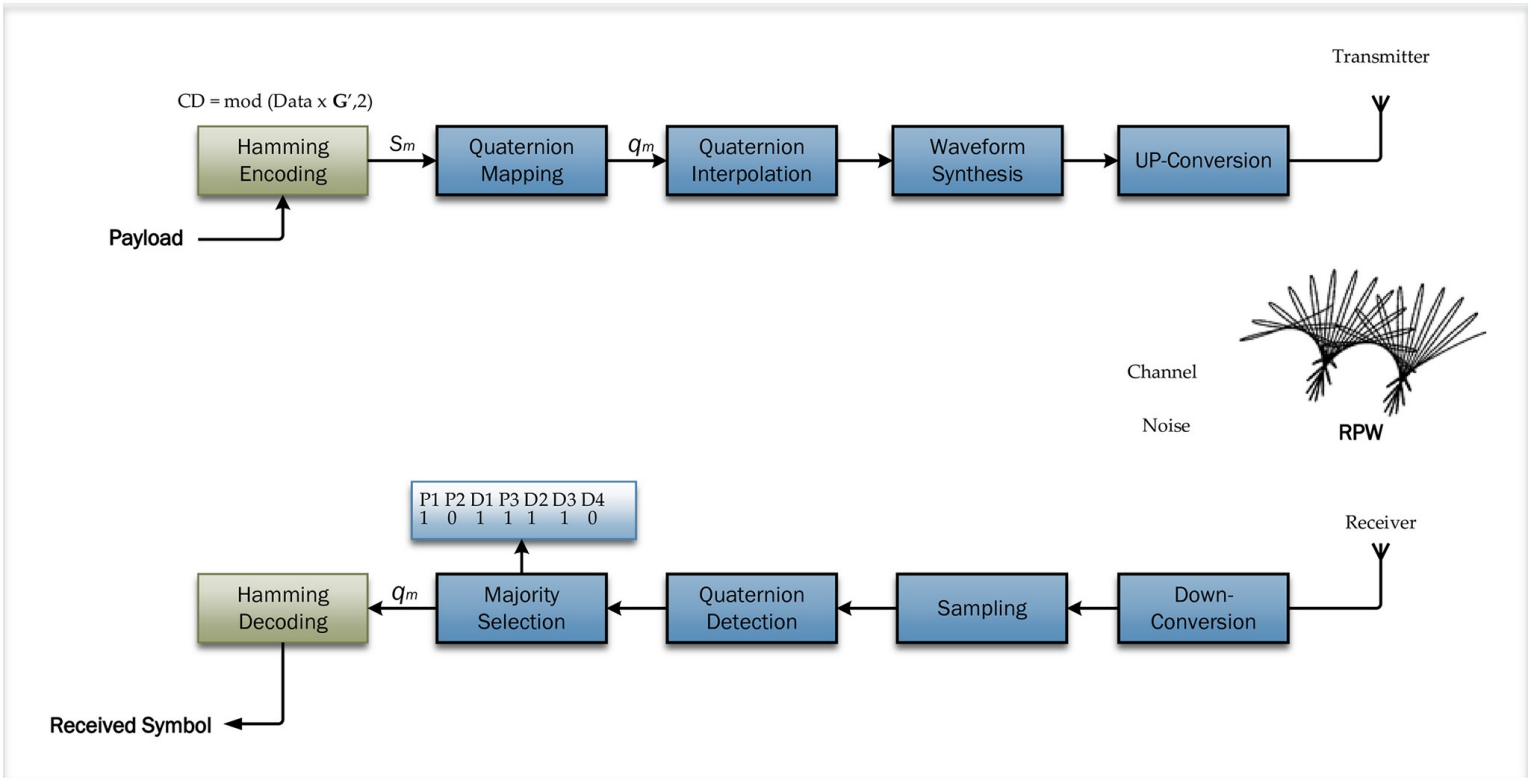
Block diagram of Hamming coded Q-RPW.

Meanwhile [28] examines the utilization of Bit Interleaved Code Modulation (BICM) to improve the performance of LoRa communication. The paper specifically emphasized the error correction capabilities of BICM in LoRa communication. The utilization of Forward Error Correction (FEC) codes in the physical layer of LoRa, is predominantly based on the basic (7,4) Hamming code with coding rates ranging from 4/5 to 4/8. The study investigated the feasibility of utilizing BICM for LoRa signals, with a focus on its capacity to optimize the coding benefits which could be achieved using the current FEC codes in commercial LoRa chips. The inquiry entailed evaluating the BER performance in both AWGN and Rayleigh fading channels. In channels affected by AWGN using BICM with a coding rate (CR) of 1 demonstrated a 40% increase in data rates. This approach achieved a BER performance comparable to that of hard decision decoding with a coding rate of 3. BICM demonstrated significant advantages in Rayleigh fading channels resulting in performance enhancements ranging from 7.2dB to 8.0dB when compared to hard decision decoding with a coding rate of 3. Significantly, BICM also exhibited significant BER improvements of 25.5 decibels when compared to the BER performance without coding in Rayleigh channels. The schematic representation of Q-RPW system model shown in Fig 3, integrates Hamming encoding and decoding techniques consists of multiple interconnected modules that collaborate to achieve resilient transmission of information. The RP-MPSK model is designed to analyze the polarization properties of electromagnetic waves in a multipath environment at the receiving antenna. The model employs quaternion representation, in which complex symbols are communicated via horizontal and vertical polarized elements in dual polarized systems. Adaptations for RP-MPSK involve introducing a phase difference of *π*/2 between base-band modulation frequencies. Quaternion algebra is utilized for the purpose of rotations in the polarization domain, and the resultant quaternion stream is transformed to a higher frequency for radio frequency transmission. After being received, the RF signal undergoes down conversion. The quaternion channel, which is characterized by real random variables, represents the received signals from both horizontal and vertical polarized antennas. The quaternion model offers the advantage of reducing the required number of real random variables by half in comparison to the standard approach. Maximum Likelihood Detection (MLD) is used to retrieve the broadcast sequence, showcasing the effectiveness of the quaternion model for RPW communication and then received data is passed through decoder for decoding of errors and removal of parity bits. During the encoding phase, the original data is subjected to quaternion rotation to modulate the polarization wave efficiently. Afterward, the data undergoes Hamming encoding which introduces redundancy to facilitate error detection and correction. The encoded blocks, which have been enhanced with supplementary parity information are transmitted across the communication channel. After being received, the RP-MSPK system proceeds to the decoding phase during which Hamming decoding is employed to detect and correct errors in the received blocks. After the decoding process, quaternion inverse rotations are applied to get the original data. The utilization of this integrated methodology guarantees the preservation of polarized wave information, while simultaneously improving the dependability and consistency of the transmitted data through the implementation of Hamming encoding and decoding. As a result, this approach is highly suitable for applications that require resilient communication in quaternion-based polarization systems.

The use of Hamming codes in this research study for Q-RPW model is based on their ability to detect and correct singe bit errors in case of Hamming (7,4) whereas in case of Hamming (15,11) can detect up to 2 errors and correct single bit errors. Since the working principle of both the (7,4) and (15,11) is almost similar, we have used Hamming (7,4).

### 3.3 Development of Hamming coded RP-MPSK and RP-QPSK

For software development of Hamming Coded RP-MPSK communication systems, the theoretical developments are converted into software implementation. The software development carried out in MATLAB consists of many components that are specifically planned to enable smooth integration with the Q-RPW framework facilitating the encoding and decoding processes. The encoding module utilizes advanced techniques to coordinate quaternion rotations on the received data. This crucial stage corresponds to the distinct attributes of RP-MPSK in which the polarization data is manipulated by means of quaternion rotations guaranteeing effective information encoding. The Hamming (7,4) coding method is used in the process to intentionally introduce redundancy which aids in robust error detection and correction as shown in the block diagram in Fig 3. The process of encoding which consists of two layers serves the purpose of encapsulating both the theoretical principles outlined in this study and establishing a practical basis for the dependable conveyance of information. The decoding module is an essential part of the software architecture which complements the communication spectrum. Customized algorithms perform reverse quaternion rotations to extract the original data from the RPW modulated signals. The decoding process is enhanced by the implementation of Hamming decoding which utilizes the redundancy included during encoding to identify and rectify any faults that may have occurred during transmission effectively. The decoding process is carefully coordinated to reconstruct the original data properly assuring the fidelity of the communicated payload. The powerful encoding and decoding software framework enhance the capabilities of Q-RPW communication system to overcome the difficulties caused by noise, interference, and other impairments in consideration of real-world situations. In addition, the software development process includes the integration of different modulation schemes into the Q-RPW framework. The software architecture smoothly incorporates RP-8PSK, RP-16PSK, and RP-QPSK modulations. The ability of various modulation schemes to allow methodically assess the performance of different levels of modulation complexity in the RPW paradigm. The software modules are intricately patched together to provide seamless transition between modulation schemes, enabling thorough comparative analysis across different channel circumstances. Being an agile and dynamic communication paradigm Q-RPW can modify its modulation approach to suit the specific demands of various real-world circumstances.

The software development is specifically designed to effortlessly connect RPW transceivers and antennas as shown in Fig 1. This comprehensive method enables hardware in the loop simulations, enabling thorough testing and validation of the proposed communication system of Fig 3. The incorporation of encoding and decoding modules along with the integration of flexible modulation schemes establishes the foundation for a resilient and versatile communication paradigm. This software designed specifically for Q-RPW communication systems allows for immediate experimentation to demonstrate the theoretical improvements discussed in this research study. Additionally, it positions RPW as a promising solution for the changing field of wireless communication wherein resilience, adaptability, and versatility in the face of various and difficult real-world situations are required. Table 2 lists the simulation parameters along with modulation type and channel type, etc. The use simulation parameters and integration of building blocks guaranteed the efficient translation of theoretical developments into software implementations.

**Table 2:** Simulation parameters used for Q-RPW model simulations.

Parameters	RPW	LoRa
No. of Symbols, Ns	100,000	100,000
No. of Monte Carlo Trails, Nt	100	100
No. of polarization samples, Np	3,5,7	-
Spreading factor for LoRa SF	-	7,9,12
SNR	1-10	1-10
Modulation	QPSK and MPSK	CSS
Error Correction Coding	Hamming (7,4)	Hamming (7,4)

## 4 Results and discussion

The simulations carried out to assess the effectiveness of the RPW communication system are discussed and greatly examined. The RP-MPSK system incorporates integrated Hamming encoding and decoding techniques as shown in Fig 3. The simulations enabled thorough examination of the effectiveness of Hamming encoding and decoding procedures in reducing errors that occur during transmission of data. A Hamming Coded LoRa communication system of Fig 2 is used to compare the results of RPW with LoRa. The resultant influence of channel impairments on the bit error rate a critical measure used to evaluate the dependability of the system has, primary been analyzed.

The observed enhancements in BER provide insight into Q-RPW system’s increased ability to withstand distortions and noise caused by the channel. The systematic analysis of simulation outcomes offers valuable insights into the concrete advantages obtained from incorporating Hamming codes into the RP-QPSK and RP-MPSK framework. This in turn informs the effectiveness of Q-RPW communication paradigm of Fig 3 in practical scenarios where maintaining low error rates is of great significance.

**Fig 4:**
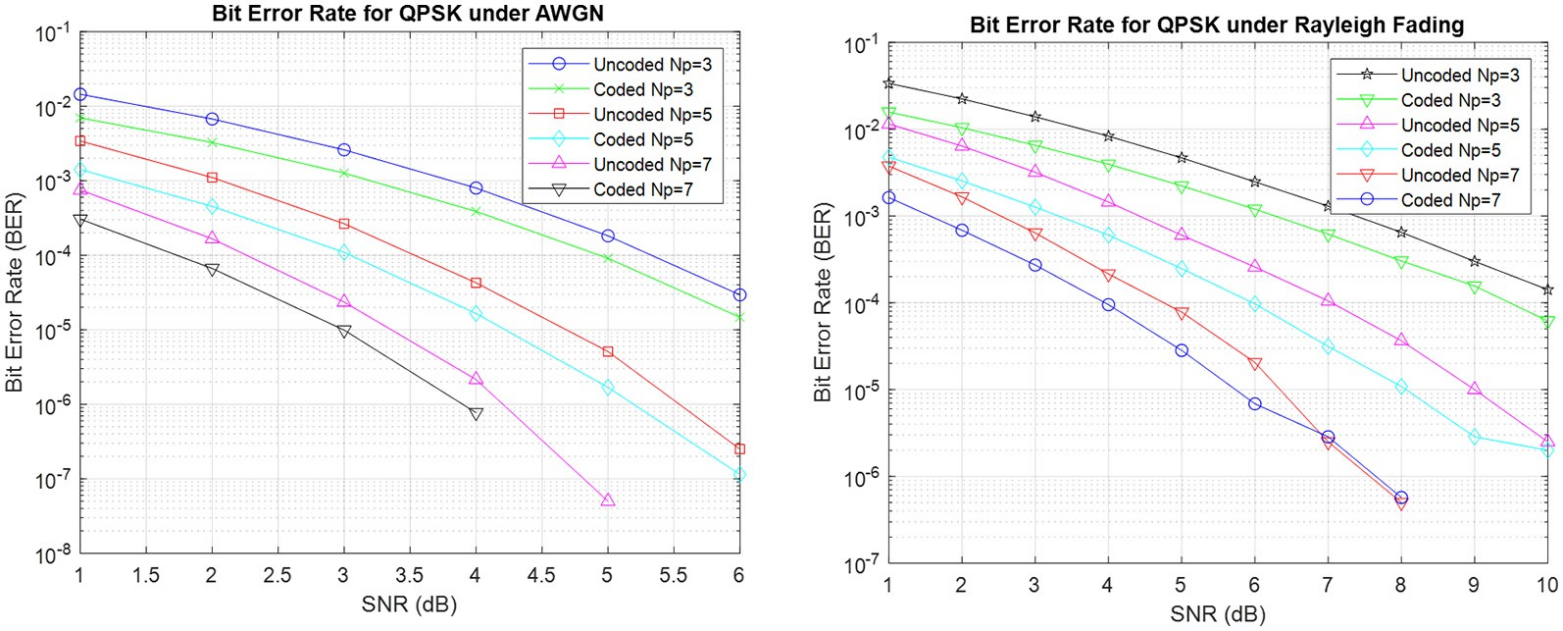
BER comparison of coded RP-QPSK under AWGN and Rayleigh fading conditions.

The findings in Fig 4 show a significant enhancement in the performance of BER for RP-QPSK systems that utilize Hamming coding, as compared to RP-QPSK systems without any coding. The utilization of Hamming codes results in a 40% improvement in withstanding errors as compared to uncoded RP-QPSK as seen by a reduced BER, thus providing support for the efficacy of error detection and correction techniques. In addition, results reveal that there is a gradual decrease in the BER when the number of polarization samples (Np) increases from Np = 3 to Np = 7. This occurrence highlights the positive effects of enhanced polarization diversity in reducing errors for improved dependability in Q-RPW communication systems. The analysis encompasses a wide range of channel conditions, where the BER in the presence of AWGN is observed to have a smaller magnitude in comparison to situations typified by Rayleigh fading conditions and AWGN has 3 dB better performance. This observation highlights the resilience of the RPW system when subjected to idealized noise levels, hence confirm-ing its suitability for reliable and accurate communication applications.

**Fig 5:**
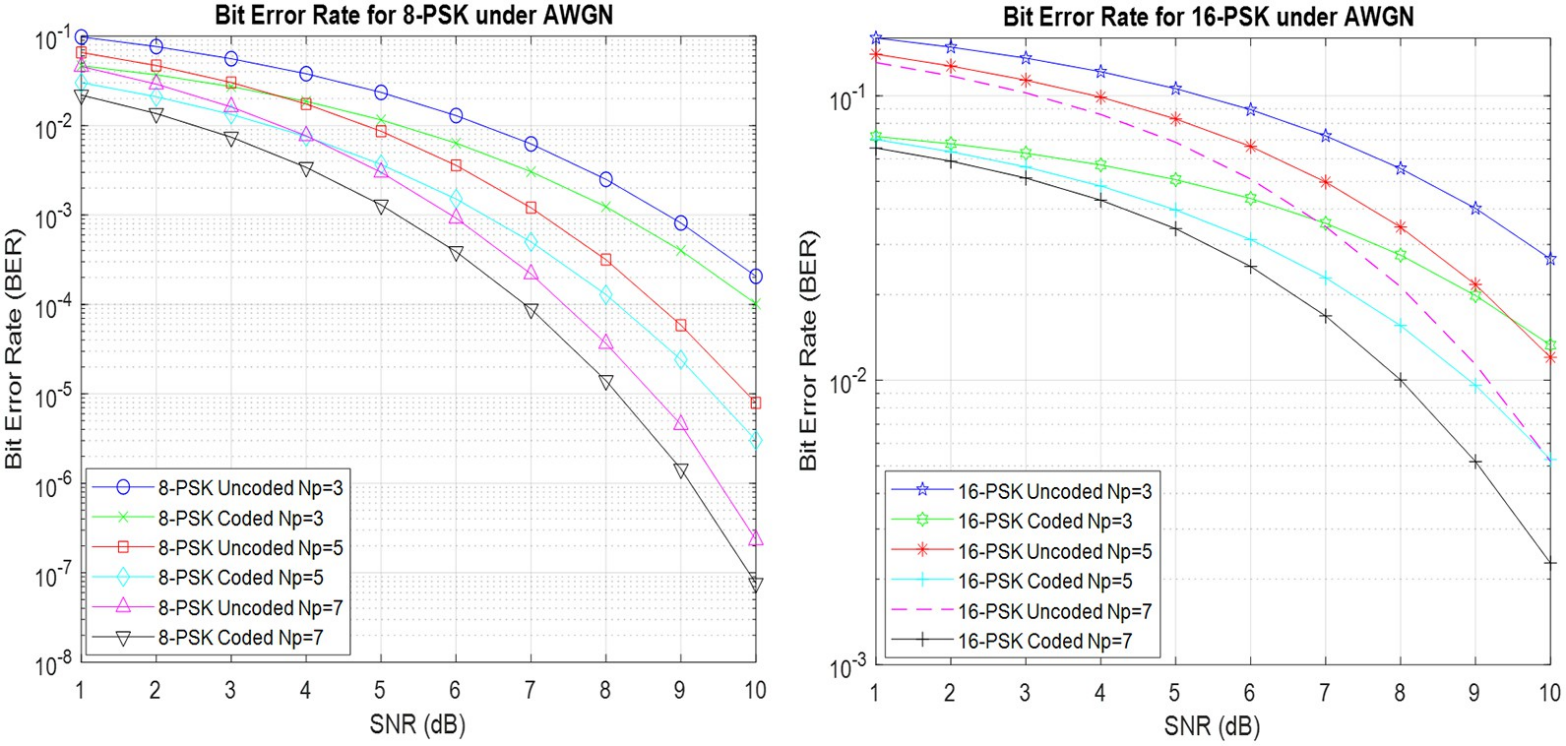
BER for coded RP-MPSK under AWGN conditions.

Utilizing RP-MPSK with Hamming coding reveals a continuous and remarkable pattern as shown in Fig 5: BER for RP-MPSK setups confirms that using Hamming coding consistently performs better than the configurations that do not use any coding. The presence of Hamming codes demonstrates the strength and durability that they bring to the data transmission process by offering a vital level of error detection and correction. In addition, when the number of polarization samples gradually grows, a simultaneous decrease in BER is observed, highlighting the significant impact of increased polarization variety in improving error tolerance. The detailed study of modulation schemes reveals a clear difference in performance between 8-PSK and 16-PSK with the former exhibiting bet-ter BER performance [13]. It can also be observed for 16-PSK the BER for coded Np = 7 is 0.9dB or almost 40% lower than the uncoded whereas for 8-PSK BER is almost 0.2dB better than the uncoded under AWGN conditions.

**Fig 6:**
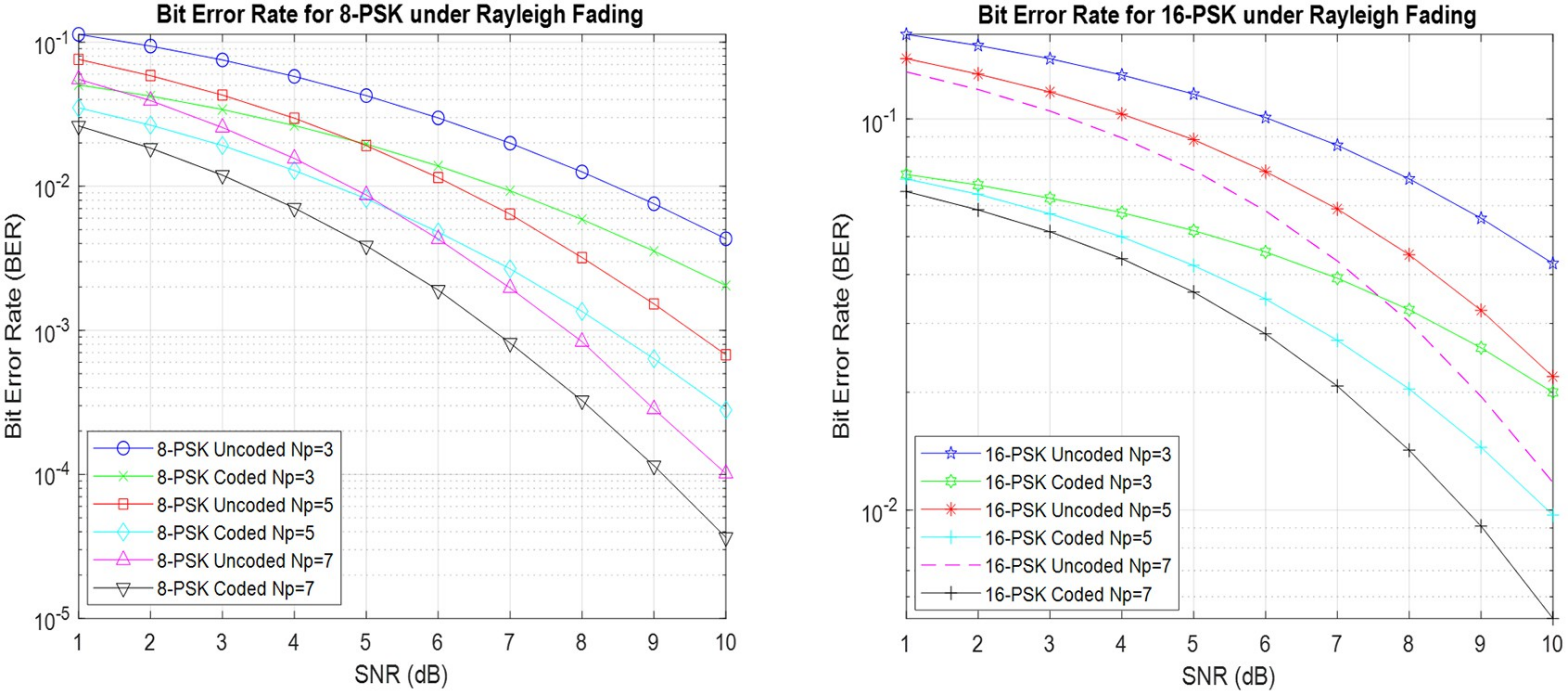
BER for Hamming(7,4) coded RP-MPSK under Rayleigh fading.

Fig 6 illustrates the effectiveness of RP-MPSK communication systems that employ Hamming (7,4) coding in combination with RP-MPSK. The noticeable improvement in BER highlights the effectiveness of the error correction methods embedded in the Hamming (7,4) code, to efficiently reduce data transmission errors. Fig 6 emphasizes on the better performance of BER in the presence of Rayleigh fading in comparison to scenarios with AWGN circumstances. Also 16-PSK with higher number of polarization samples provides 1.1dB or 48% better results in BER performance. The difference highlights the ability of the Hamming (7,4) coded RP-MPSK configuration to withstand dynamic channel limitations. Corrections in the Hamming (7,4) code are clearly shown in the displayed bit error rate, which provide an essential understanding of how coding methods and channel characteristics interact in RPW communication systems.

**Fig 7:**
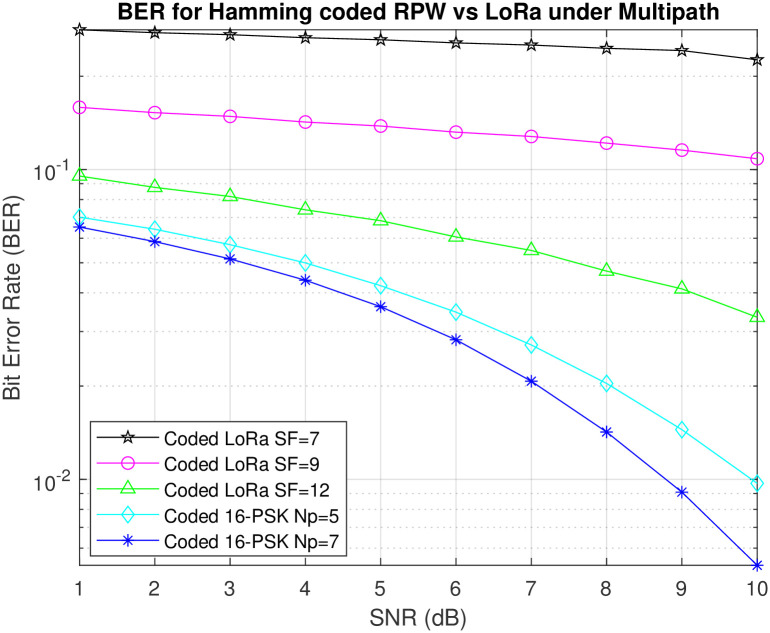
Comparison between Hamming coded LoRa and Q-RPW.

The communication paradigms involving RPW and LoRa are compared to determine their effectiveness in different environmental settings. Fig 7 illustrates our examination of the performance of RP-MPSK and RP-QPSK compared to LoRa in difficult multipath situations with Rayleigh Fading. The results of our study reveal a significant benefit for RPW, especially when combined with Hamming coding. Hamming codes have greatly enhanced the robustness of RPW communication, leading to greater performance in comparison to LoRa. LoRa utilizes Chirp Spread Spectrum (CSS) modulation, which is widely used for long distance communication. However, our findings show that RPW with 8-PSK, 16-PSK, and QPSK modulations is highly resilient for long distance communication. The analysis of complete results highlights the clear benefits of RPW compared to LoRa in reducing the negative impact of multipath propagation, providing a promising solution for communication systems in challenging environments. In addition, the results provide useful insights into the complex interaction among modulation schemes, coding strategies, and channel characteristics for the design and implementation of Q-RPW communication systems for real world applications.

The simulation of RPW communication systems, specifically in combination with Hamming coding and RP-MPSK modulations, provides valuable insights to complement prior research and the fundamental hypotheses. The advantage of Hamming (7,4) coded RP-MPSK topologies, as depicted in Figs 4–7 corresponds with existing understanding in error correction coding. The notable improvement in BER after applying single bit error corrections confirms the efficacy of the Hamming code in reducing data transmission thus supporting our hypothesis about the resilience of error correction mechanisms in quaternion-based communication systems. Fig 7 showed higher performance of RP-MPSK and RP-QPSK when compared to LoRa employing CSS in multipath scenarios. Error performance of coded RPW outperforms coded CSS modulation used in LoRa under multipath conditions by 51%, demonstrating superior adaptability and robustness under dynamic channel conditions. This aligns with the expected benefits of RP-MPSK in reducing the effects of dynamic channel impairments supporting our initial theory that RP-MPSK utilizes distinct polarization-based information carriers to provide greater durability in demanding communication situations as compared to standard modulation schemes like CSS. These findings have ramifications for designing and implementing communication systems in real-world scenarios, especially those with difficult channel conditions.

Table 3 provides a detailed overview of the performance of various modulation schemes within RPW communication systems, highlighting improvements in BER under different channel conditions. The comparisons with LoRa emphasize the quantitative advantages of Quaternion RPW over it while the impact of Hamming coding on error resilience is elucidated for each modulation scheme. The combination of RPW, Hamming Coding, and multiple MPSK modulations enhances its applicability in scenarios where reliability, minimal error rates, and adaptation to different environmental obstacles are essential.

**Table 3:** Summary of results in Hamming coded Q-RPW communication system.

Modulation Scheme	AWGN Conditions	Rayleigh Fading	AWGN BER Improvement (Hamming Coded RPW vs. Uncoded)	Rayleigh Fading BER Improvement (Hamming Coded RPW vs. Uncoded)
**RP-QPSK**	Significant improvement in BER.	Superior performance compared to other modulations under Rayleigh Fading.	Substantial (40%) improvement with Hamming coding, affirming its efficacy.	Outperforms uncoded QPSK by 42%, highlighting the value of Hamming coding.
**8-PSK**	Substantial improvement in BER.	Demonstrates superior performance.	Significant improvement (0.2dB) with Hamming coding, enhancing error resilience.	Superior (0.4dB) performance with Hamming coding, showcasing robustness.
**16-PSK**	Moderate improvement in BER.	Exhibits enhanced resilience under Rayleigh Fading.	Moderate (0.9dB) improvement observed with Hamming coding.	Enhanced (1.1dB) resilience in Rayleigh Fading conditions with Hamming coding.
**LoRa vs. Q-RPW**	LoRa demonstrates quantifiable improvement in BER under AWGN conditions compared to RPW [5].	RPW consistently outperforms LoRa, affirming its suitability for robust communication.	N/A	RPW with 16-PSK performs better than LoRa under Rayleigh fading.

In this study, Hamming codes offered a good balance between error correction capability and decoding complexity while attempting to significantly preserve the integrity of the data. The future research into RPW systems lies in the comparative analysis of error correction techniques and their impact on overall energy efficiency. The effectiveness of Hamming codes in RPW systems alongside alternative coding schemes can be investigated to obtain valuable insights into optimizing energy consumption whilst maintaining reliable data transmission. This endeavor can consider possible factors such as code rate, decoding power requirements, and the resulting impact on transmission power levels required to achieve targeted BER to cater for the advancements of quaternion-based communication paradigm. In addition, the coverage of RPW through hardware implementation for large scale networks demands further focus on refining the parameters of Q-RPW configurations by examining new coding schemes and assessing their scalability for real-world applications.

## 5 Conclusions

The methodical investigation of Q-RPW communication model, enhanced with Hamming coding and diverse QPSK and MPSK modulations, has produced valuable observations with significance for the wider field of wireless communication for real time applications in Industrial IoT monitoring and Healthcare Telemetry. The effectiveness of Hamming (7,4) coding in improving the ability of RP-MPSK setups to withstand errors matches with a well established understanding, confirming the strength of error correcting mechanisms in quaternion-based communication systems. The demonstrated superiority of Q-RPW over LoRa in the presence of multipath situations highlights the potential benefits of Q-RPW in stringent communication environment. These findings add to the ongoing research on the design of communication systems, highlighting the importance of quaternion-based techniques in terms of their flexibility and dependability. This study establishes a basis for comprehending RPW communication systems in various scenarios involving error correction coding. The exceptional efficacy of Hamming coded Q-RPW, especially in reducing errors and adjusting to difficult channel conditions, establishes this communication paradigm as a potential option for practical applications where dependability and flexibility are of supreme importance and our research not only validates the theoretical underpinnings of Hamming-coded Q-RPW but also quantifies its advantageous performance compared to LoRa. Further research work is undertaken on optimizing Q-RPW parameters by incorporating new coding schemes and examining the scalability of the proposed communication paradigm in large scale networks.

## References

[1] Shahgholi T, Sheikhahmadi A, Khamforoosh K, Azizi S. LPWAN-based hybrid backhaul communication for intelligent transportation systems: architecture and performance evaluation. EURASIP J Wirel Commun Netw. 2021 Dec 1;2021(1). 2. doi: 10.1186/s13638-021-01918-2

[2] Islam N, Ray B, Pasandideh F. IoT Based Smart Farming: Are the LPWAN Technologies Suitable for Remote Communication? In: 2020 IEEE International Conference on Smart Internet of Things (SmartIoT). IEEE; 2020. p. 270–6.

[3] Mekki K, Bajic E, Chaxel F, Meyer F. A comparative study of Lpwan Technologies for large scale IOT deployment. ICT Express. 2019 Mar;5(1):1–7. doi: 10.1016/j.icte.2017.12.005

[4] Ali MM, Hashim SJ, Chaudhary MA, Ferré G, Rokhani FZ, Ahmad Z. A Reviewing Approach to Analyze the Advancements of Error Detection and Correction Codes in Channel Coding With Emphasis on LPWAN and IoT Systems. IEEE Access. 2023;11:127077–97. doi: 10.1109/ACCESS.2023.3331417

[5] Ahmad Z, Hashim SJ, Ferre G, Rokhani FZ, Al-Haddad SAR, Sali A. LoRa and Rotating Polarization Wave: Physical Layer Principles and Performance Evaluation. IEEE Access. 2023;11:14892–905. doi: 10.1109/ACCESS.2023.3242552

[6] Peppas K, Chronopoulos SK, Loukatos D, Arvanitis K. New results for the error rate performance of Lora systems over fading channels. Sensors. 2022 Apr 27;22(9):3350. doi: 10.3390/s22093350 35591038 PMC9101127

[7] Courjault J, Vrigneau B, Berder O. Fast performance evaluation of lora communications over Rayleigh Fading Channels. 2019 IEEE Wireless Communications and Networking Conference Workshop (WCNCW). 2019 Apr;

[8] Coutaud U, Heusse M, Tourancheau B. Fragmentation and Forward Error Correction for LoRaWAN Small MTU Networks. In: Proceedings of the 2020 International Conference on Embedded Wireless Systems and Networks. USA: Junction Publishing; 2020. p. 289–94. (EWSN’20).

[9] Elzeiny S, Edward P, Elshabrawy T. LoRa Performance Enhancement through List Decoding Technique. In: 2021 IEEE International Conference on Communications Workshops (ICC Workshops). IEEE; 2021. p. 1–6.

[10] Zhao Zahi Wei, Min Geyong, Gao Weifeng, Mo Jiwei, Huan Gwenjie. Error detection and adaptive error correction method based on LoRa coding and decoding mechanism. China; CN109525367B, 2021.

[11] Yamada H, Takei K. Highly-Reliable Rotating Polarization Wave Transceiver with Optimal Polarization Selection. In: 2019 IEEE Radio and Wireless Symposium (RWS). IEEE; 2019. p. 1–3.

[12] Takei K, Yamada H. Highly reliable wireless communication system using polarization rotating wave for M2M application. Electronics and Communications in Japan. 2019 Mar 22;102(3):29–35. doi: 10.1002/ecj.12146

[13] Ahmad Z, Hashim SJ, Rokhani FZ, Al-Haddad SAR, Sali A, Takei K. Quaternion Model of Higher-Order Rotating Polarization Wave Modulation for High Data Rate M2M LPWAN Communication. Sensors. 2021 Jan 7;21(2):383. doi: 10.3390/s21020383 33430523 PMC7826550

[14] Warren L. Stutzman. Polarization in Electromagnetic Systems. Second Edition. Boston, MA, USA,: Artech House; 2018.

[15] Gali S, Wauer E, Nikoubin T. Low Power and Energy Efficient Single Error Correction Code using CDM logic style for IoT devices. In: 2018 IEEE 13th Dallas Circuits and Systems Conference (DCAS). IEEE; 2018. p. 1–5.

[16] Ahmad Z, Hashim SJ, Rokhani FZ, Al-Haddad SAR, Sali A. Enhanced Rotating Polarization Wave for Robust Wireless Connectivity. In: 2020 9th IFIP International Conference on Performance Evaluation and Modeling in Wireless Networks (PEMWN). IEEE; 2020. p. 1–6.

[17] Lin S, Costello DJ. Error control coding: Fundamentals and applications. Noida, India: Pearson India; 2011.

[18] Chen W, Zhao T, Han C. Soft decision decoding with cyclic information set and the decoder architecture for cyclic codes. Electronics. 2023 Jun 16;12(12):2693. doi: 10.3390/electronics12122693

[19] Bere PS, Khan MZ. Bit switching decoding of cyclic hamming codes for IOT applications. 2022 IEEE World AI IoT Congress (AIIoT). 2022 Jun 6;

[20] Robert H. Morelos-Zaragoza. The Art of Error Correcting Coding. 2nd ed. John Wiley & Sons; 2006.

[21] Chlaab AK, Flayyih WN, Rokhani FZ. Lightweight hamming product code based multiple bit error correction coding scheme using shared resources for on chip interconnects. Bulletin of Electrical Engineering and Informatics. 2020 Oct 1;9(5):1979–89. doi: 10.11591/eei.v9i5.1876

[22] Alom MdR, Shakib MdN, Rahaman MA. Enhanced hamming codes: Reducing redundant bit for efficient error detection and correction. 2023 5th International Conference on Sustainable Technologies for Industry 50 (STI). 2023 Dec 9;

[23] Chen Z, Zhao Y, Lu J, Liang B, Chen X, Li C. Teced: A two-dimensional error-correction codes based energy-efficiency SRAM design. Electronics. 2022 May 20;11(10):1638. doi: 10.3390/electronics11101638

[24] Alnajjar KA, Abushawish AYI, Ansari S. Hardware-Based Error Correction Systems for Hamming Codes: a Review of the Literature. In: 2023 International Conference on Smart Applications, Communications and Networking (SmartNets). IEEE; 2023. p. 1–8.

[25] Hussain GA, Wahhab HA, Harbi YJ. Filtered OFDM System Improvement Using Hamming Code. Journal of Communications. 2023. doi: 10.12720/jcm.18.4.215-220

[26] Xie H, Qi Y, Alyousuf FQA. Designing an ultra-efficient Hamming code generator circuit for a secure nano-telecommunication network. Microprocess Microsyst. 2023 Nov;103:104961. doi: 10.1016/j.micpro.2023.104961

[27] Afisiadis O, Burg A, Balatsoukas-Stimming A. Coded LoRa Frame Error Rate Analysis. In: ICC 2020—2020 IEEE International Conference on Communications (ICC). IEEE; 2020. p. 1–6.

[28] Elshabrawy T, Robert J. Evaluation of the BER Performance of LoRa Communication using BICM Decoding. In: 2019 IEEE 9th International Conference on Consumer Electronics (ICCE-Berlin). IEEE; 2019. p. 162–7.

