# Correction: Error mitigation in LPWAN systems: A study on the efficacy of Hamming-coded RPW

**DOI:** 10.1371/journal.pone.0323009

**Published:** 2025-04-22

**Authors:** Muhammad Moazzam Ali, Shaiful Jahari Hashim, Zaid Ahmad, Guillaume Ferre, Fakhrul Zaman Rokhani, Muhammad Akmal Chaudhary

The first author, Muhammad Moazzam Ali, is incorrectly noted as the corresponding author. The correct corresponding author is Shaiful Jahari Hashim. Shaiful Jahari Hashim’s email address is: sjh@upm.edu.my.
